# Occurrence and Antimicrobial Resistance Profiles of Culturable Ampicillin-Resistant Gram-Negative Bacteria in the Ring of Cenotes Aquifer, Yucatán, Mexico

**DOI:** 10.3390/microorganisms14091903

**Published:** 2026-08-28

**Authors:** Patricia Vargas-Gutiérrez, José Augusto Ramírez-Trujillo, Ana Busto-Ulloa, Luis Lozano-Aguirre, Juan Téllez-Sosa, Paola Bocanegra-Ibarias, Gabriel Lizama-Uc, Ismael Hernández-Lucas, Ramón Suárez-Rodríguez, Josefina Duran-Bedolla, Humberto Barrios-Camacho

**Affiliations:** 1 Facultad de Ciencias Biológicas, Doctorado en Ciencias Naturales, Universidad Autónoma del Estado de Morelos (UAEM), Cuernavaca 62209, Morelos, Mexico; 2 Centro de Investigación en Biotecnología, Universidad Autónoma del Estado de Morelos (UAEM), Cuernavaca 62209, Morelos, Mexico; 3 Unidad de Análisis Bioinformáticos, Centro de Ciencias Genómicas, Universidad Nacional Autónoma de México, Cuernavaca 62210, Morelos, Mexico; 4 Centro de Investigación Sobre Enfermedades Infecciosas (CISEI), Instituto Nacional de Salud Pública (INSP), Cuernavaca 62100, Morelos, Mexico; 5 Departamento de Enfermedades Infecciosas, Hospital Universitario “Dr. José Eleuterio González”, Facultad de Medicina, Universidad Autónoma de Nuevo León, Monterrey 64460, Nuevo León, Mexico; 6 Unidad de Posgrado e Investigación, Tecnológico Nacional de México/IT Mérida, Merida 97000, Yucatán, Mexico; 7 Instituto de Biotecnología, Universidad Nacional Autónoma de México, Cuernavaca 62210, Morelos, Mexico

**Keywords:** *E. coli*, AMR, sinkhole, environment

## Abstract

Antimicrobial resistance (AMR) is one of the main public health problems that also affects environmental ecosystems. Sinkholes are understudied groundwater ecosystems that may act as reservoirs and dissemination routes for AMR. This study evaluated the composition, distribution, and antimicrobial resistance profiles of culturable ampicillin-resistant Gram-negative bacteria recovered from sinkholes of the Yucatán Peninsula, Mexico. Water samples were collected from 27 sinkholes during dry and rainy seasons. The isolates were identified by MALDI-TOF MS and evaluated by broth microdilution. Representative *E. coli* isolates were analyzed by whole-genome sequencing. A total of 193 ampicillin-resistant Gram-negative isolates were recovered. The most frequently recovered genera were *Enterobacter*, *Klebsiella*, and *Escherichia*, which were detected across all hydrogeological zones. Resistance to cefotaxime, levofloxacin, gentamicin, and tetracycline was detected among several bacterial genera. Opportunistic bacterial species associated with healthcare-associated infections, including *K. pneumoniae*, *A. baumannii*, and *P. aeruginosa*, were also identified. Genomic analysis of 19 *E. coli* isolates revealed resistance determinants associated with multiple antimicrobial classes and a diverse population structure dominated by phylogroup B1. These findings show that the Ring of Cenotes aquifer harbors diverse populations of culturable ampicillin-resistant Gram-negative bacteria and emphasize the importance of groundwater ecosystems as environmental reservoirs of AMR.

## 1. Introduction

Antimicrobial resistance (AMR) is recognized as one of the most important global threats to public health. The World Health Organization (WHO) has identified antibiotic-resistant bacteria as a major challenge for the treatment of infectious diseases. The WHO emphasized the need to address AMR from a One Health perspective that integrates human, animal, and environmental health [1]. Although the clinical consequences of AMR are well-documented, increasing evidence indicates that natural and human-impacted environments play a critical role in the maintenance and dissemination of resistant bacteria and antimicrobial resistance genes (ARGs) [2,3].

Aquatic ecosystems are increasingly recognized as environmental reservoirs of AMR because they receive microorganisms and contaminants from multiple sources, including wastewater discharges, agricultural activities, livestock production, and urban runoff [4,5]. The WHO specifically prioritizes the study and identification of the Gram-negative bacteria associated with the spread of extended-spectrum β-lactamases (ESBLs) and carbapenemases [6].

Karst aquifers represent a vulnerable environment for the study of AMR dissemination. Their high permeability, extensive fracture networks, and limited natural filtration facilitate the rapid infiltration of contaminants from surface environments into groundwater systems [7]. These characteristics distinguish karst groundwater from more contained anthropogenic environments because resistant bacteria and contaminants introduced at the surface can quickly enter and disperse through a highly connected aquifer system.

The Yucatán Peninsula, Mexico, contains one of the largest karst aquifer systems in the world and depends almost entirely on groundwater as its principal freshwater resource [8]. In this region, the Ring of Cenotes constitutes a hydrogeological structure associated with the Chicxulub impact crater and is characterized by extensive groundwater connectivity [9]. Previous investigations have documented the presence of fecal contamination, chemical pollutants, and antimicrobial resistance genes in the sinkholes of the Yucatán Peninsula [10,11,12,13]. However, information regarding the occurrence, diversity, and resistance profiles of culturable antibiotic-resistant Gram-negative bacteria across different hydrogeological zones of this aquifer remains limited.

The objective of this study was to characterize the occurrence and antimicrobial resistance profiles of culturable ampicillin-resistant Gram-negative bacteria recovered from sinkholes distributed throughout the Ring of Cenotes aquifer. We also investigated whether the occurrence of resistant bacteria varied among hydrogeological zones and between the dry and rainy seasons.

## 2. Material and Methods

### 2.1. Study Area and Sampling

**Figure 1:**
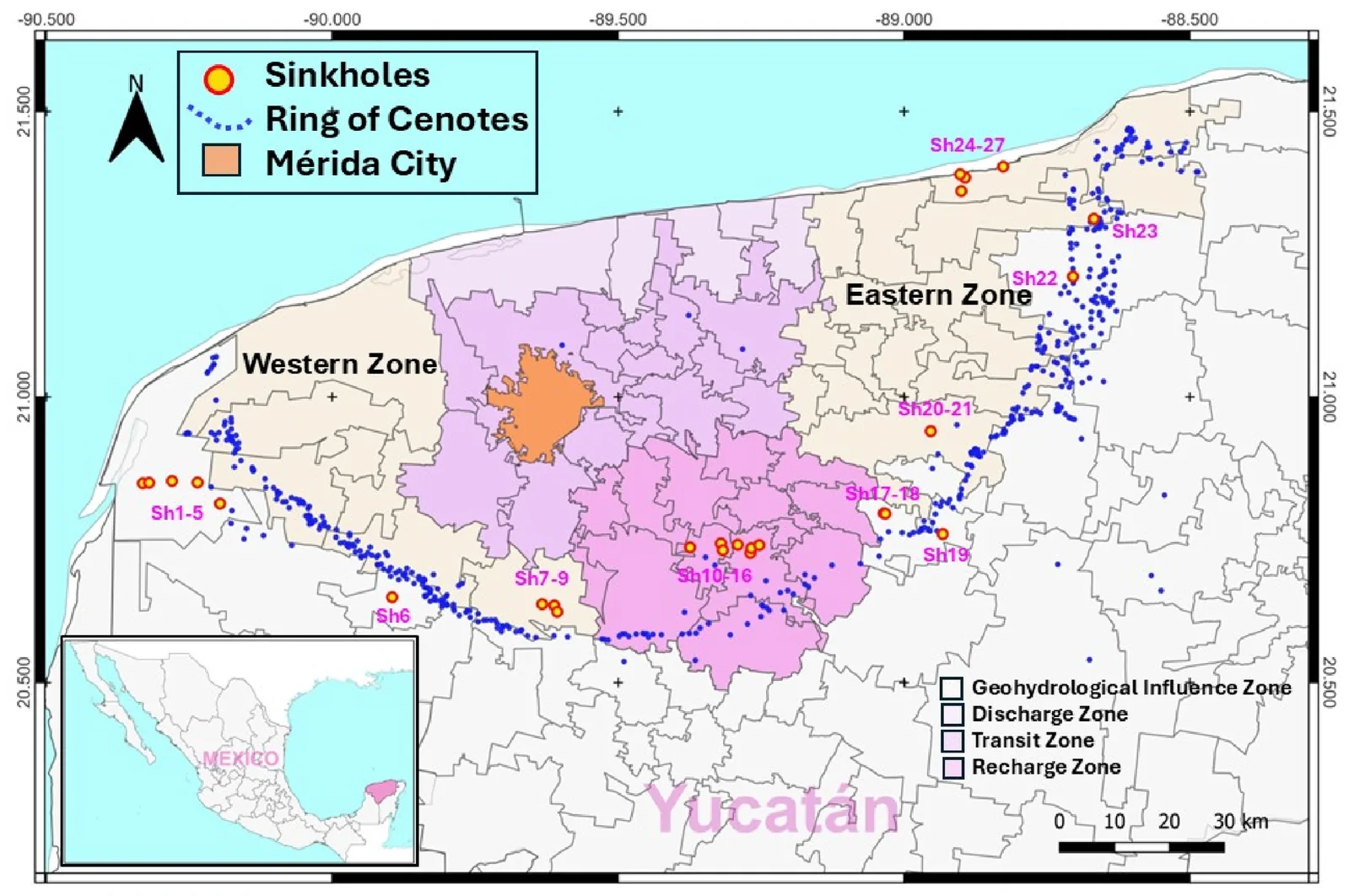
Geographic distribution of the 27 sinkholes sampled across the hydrogeological zones of the Ring of Cenotes aquifer, Yucatán, Mexico.

This study was conducted in the Ring of Cenotes, a Geohydrological Reserve in Yucatán, Mexico. This karst aquifer is associated with the Chicxulub impact structure and is characterized by high hydraulic connectivity, rapid infiltration, and continuous groundwater flow. In the sinkholes, water circulates between inflow, transition, and outflow sectors. To account for this hydrodynamic variability, three water subsamples were collected from each sinkhole, and subsequently pooled to obtain a representative mixture sample for bacterial isolation. The three subsamples were considered components of a single combined sample and were not treated as independent biological replicates. A total of 27 sinkholes distributed across the Ring of Cenotes were included throughout the study period. The same 27 sinkholes were sampled during the dry (February 2023) and rainy (September 2023) seasons. Sampling sites were distributed among three hydrogeological zones previously described for the region: the Recharge zone (*n* = 7), the Western zone (*n* = 9), and the Eastern zone (*n* = 11). The spatial distribution of the sampling sites is shown in Figure 1, and the geographic coordinates and hydrogeological classification of each sinkhole are provided in Supplementary Table S1.

Water samples (250 mL) were collected in sterile containers at approximately 30 cm below the water surface in triplicate at each site and transported under refrigerated conditions for immediate laboratory processing.

### 2.2. Bacteria Isolation and Identification

The three water samples collected at each sampling site were pooled to obtain a composite sample, from which 100 mL was used for bacteria isolation. Samples were filtered through 0.45 µm membrane filters (Millipore, Billerica, MA, USA). Each membrane was transferred to 1 mL of Luria–Bertani (LB) broth and agitated for 10 min at room temperature to release retained bacteria. Subsequently, 20 µL of the resulting suspension was inoculated into 1 mL LB broth and incubated at 37 °C for 1 h. After incubation, the samples were plated onto two LB and two MacConkey agar plates, all supplemented with ampicillin (10 µg/mL). Serial dilutions were not performed because the objective was to recover representative ampicillin-resistant isolates instead of quantifying bacterial abundance. Colonies displaying distinct morphologies, including differences in colony size, shape, elevation, margin, and pigmentation, were selected. Two representative colonies from each morphotype were purified by repeated streaking on fresh agar plates until pure cultures were obtained. Pure cultures were preserved in LB broth containing 30% glycerol at −70 °C until further analysis. Species identification was performed using matrix-assisted laser desorption/ionization time-of-flight mass spectrometry (MALDI-TOF MS) (Microflex LT, Bruker Daltonics, Bremen, Germany) at the Clinical Microbiology Laboratory of the Hospital Universitario “Dr. José Eleuterio González”, Universidad Autónoma de Nuevo León, using the manufacturer’s standard identification procedures. Identification scores ≥ 2.0 were considered reliable for species-level assignment according to the manufacturer’s recommendations. Isolates identified as *Acinetobacter baumannii* were further confirmed by PCR amplification of the intrinsic *bla*OXA-51-like gene [14].

### 2.3. Antimicrobial Susceptibility Testing

Antimicrobial susceptibility was determined by broth microdilution according to Clinical and Laboratory Standards Institute (CLSI) guidelines. The antimicrobial agents evaluated included β-lactams (ampicillin and cefotaxime) and a carbapenem (imipenem), quinolones (nalidixic acid and levofloxacin), an aminoglycoside (gentamicin), a sulfonamide (sulfamethoxazole), and a tetracycline (tetracycline). Minimum inhibitory concentration (MIC) values were interpreted using organism-specific criteria from CLSI M100, 32nd edition (2022), when applicable [15]. Organism antimicrobial combinations for which no applicable CLSI M100 interpretive criteria were available were not assigned categorical susceptibility classifications. Intrinsic or expected resistance was considered according to CLSI recommendations and excluded from the classification of acquired resistance. For descriptive purposes, isolates exhibiting acquired resistance to three or more antimicrobial classes were classified according to previously proposed criteria [16]. The organism-specific interpretive criteria, intrinsic resistance, and antimicrobial agents excluded from categorical interpretation are summarized in Supplementary Table S2. *Escherichia coli* ATCC 25922 was used as the quality-control strain according to CLSI recommendations [15].

### 2.4. Whole-Genome Sequencing and Genomic Analysis of Escherichia coli

Nineteen *E. coli* isolates were selected for whole-genome sequencing to complement the phenotypic characterization of the bacterial collection. Isolates were selected to represent different antimicrobial resistance phenotypes, hydrogeological zones, and sampling seasons. Because the primary objective of this study was to characterize the occurrence and antimicrobial resistance profiles of culturable ampicillin-resistant Gram-negative bacteria, the genomic analysis was planned to provide complementary information rather than a comprehensive population genomic analysis. *E. coli* is widely recognized as an indicator of fecal contamination and is frequently used as a sentinel organism in environmental surveillance of antimicrobial resistance.

Genomic DNA libraries were prepared using the Illumina DNA Prep kit (Illumina, San Diego, CA, USA) and sequenced on an Illumina NextSeq 550 platform using paired-end chemistry. Raw sequencing reads were quality assessed using FastQC v0.12.1 and trimmed with Trim Galore v0.6.10 to remove low-quality bases and adapter sequences. The trimmed reads were then assembled using SKESA v.2.2 [17]. Assembly quality was assessed using CheckM v1.2.2, and only assemblies with ≥90% completeness and ≤5% contamination were used for subsequent analyses; genome quality metrics are provided in Supplementary Table S3 [18]. Genome annotation was performed using the NCBI Prokaryotic Genome Annotation Pipeline (PGAP). Phylogroups were assigned using the Clermont Typing platform, and multilocus sequence types (MLST) were determined using MLST 2.0 from the Center for Genomic Epidemiology. Antimicrobial resistance determinants were identified using Resistance Gene Identifier (RGI) v6.0.3 with CARD v3.2.9 [19] using the default CARD model-based criteria. Under these settings, Perfect and Strict hits were retained, while Loose hits were excluded. Intrinsic resistance-associated determinants were excluded from the comparative ARG presence/absence analysis.

## 3. Results

### 3.1. Distribution of Gram-Negative Bacteria Across Hydrogeological Sinkholes

**Figure 2:**
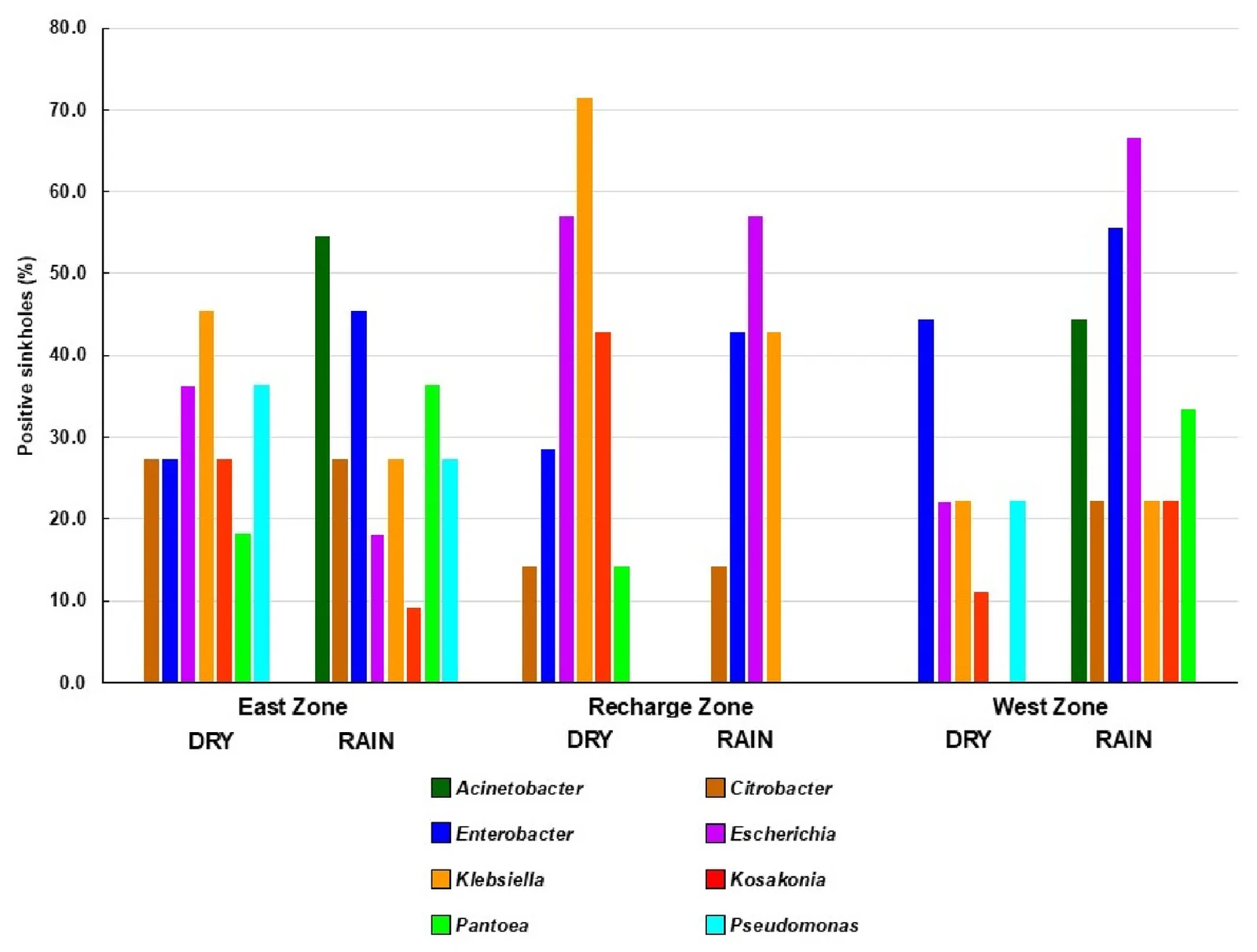
Percentage of sinkholes positive for the most frequently recovered culturable ampicillin-resistant Gram-negative bacterial genera across the hydrogeological zones during the dry and rainy seasons.

A total of 227 bacterial isolates were recovered from the 54 water samples collected throughout the study period, of which 193 corresponded to culturable ampicillin-resistant Gram-negative bacteria. A total of 69 culturable ampicillin-resistant Gram-negative isolates were recovered during the dry season, whereas 124 were recovered during the rainy season. When the occurrence of the most common genera was evaluated using sinkholes as the sampling unit, all hydrogeological zones contained sinkholes positive for one or more of the dominant bacterial genera. The frequency of positive sinkholes varied among genera and seasons, with *Enterobacter*, *Escherichia*, and *Klebsiella* showing the highest occurrence during the rainy season, particularly in the Recharge and Western zones (Figure 2).

Members of the order Enterobacterales dominated the recovered bacteria community, with *Enterobacter* (*n* = 41), *Klebsiella* (*n* = 29), and *Escherichia* (*n* = 26) representing 50.2% of all isolates recovered across hydrogeological zones and sampling periods. Other frequently recovered genera included *Pantoea* (*n* = 18), *Acinetobacter* (*n* = 16), *Citrobacter* (*n* = 9), *Kosakonia* (*n* = 9), *Pseudomonas* (*n* = 8), and *Proteus* (*n* = 6).

Several species recognized as opportunistic human pathogens were recovered throughout the study area, such as members of the *Enterobacter cloacae* complex (predominantly identified as *E. hormaechei*) (*n* = 29), *E. coli* (*n* = 26), members of the *Klebsiella oxytoca* complex (*n* = 12), and *K. pneumoniae* (*n* = 10), all of which were detected in multiple hydrogeological zones and during both sampling periods. Additional clinically relevant species included *Acinetobacter baumannii* (*n* = 6), *Serratia marcescens* (*n* = 4), and *Pseudomonas aeruginosa* (*n* = 3).

Environmental and plant-associated genera like *Pantoea*, *Kosakonia*, and *Agrobacterium* were recovered from sinkholes, and genera commonly associated with aquatic habitats or fecal contamination, such as *Aeromonas* and *Proteus*, were also identified across multiple hydrogeological zones.

Gram-positive bacteria were also recovered from several sinkholes; however, because the primary objective of this study was focused on ampicillin-resistant Gram-negative bacteria, these isolates were not included in subsequent analyses.

### 3.2. Antimicrobial Resistance Profiles

All isolates were recovered on ampicillin-supplemented media, consistent with the selective design of the study. Antimicrobial susceptibility was interpreted for organism–antimicrobial combinations for which applicable CLSI M100 criteria were available. Resistance to cefotaxime and tetracycline was observed across several bacterial species, whereas resistance to gentamicin and levofloxacin was less frequent (Table 1). No acquired resistance to imipenem was detected among the isolates for which CLSI interpretive criteria were applicable. Sulfamethoxazole and nalidixic acid were also evaluated; however, these agents were not interpreted when applicable CLSI M100 criteria were unavailable. Resistance involving multiple antimicrobial classes was identified among several members of the Enterobacterales and other Gram-negative bacteria, after excluding intrinsic or expected resistance.

### 3.3. Genomic Characterization of Environmental E. coli Isolates

Whole genome sequencing of 19 representative *E. coli* isolates revealed substantial genetic diversity, with predominant phylogroup B1 at 47.4% (9/19), followed by B2 (3/19), E (3/19), A (2/19), D (1/19), and G (1/19). Multilocus sequence typing identified 16 distinct sequence types among the 19 isolates, with only ST1204, ST3759, and ST10068 represented by more than one isolate. Whole-genome sequencing identified resistance determinants associated with β-lactams, quinolones, aminoglycosides, trimethoprim, sulfonamides, and tetracyclines (Table 2). Genes associated with β-lactam resistance included *bla*EC-5, *bla*EC-8, *bla*EC-15, *bla*EC-18, and *bla*TEM-135, whereas quinolone resistance determinants were represented by *qnrS1* and *qnrB7*. Additional resistance genes included *aadA, aadA5, APH(3′)-lb* and *APH(6)-ld* (aminoglycosides), *dfrA15* and *dfrA17* (trimethoprim), *sul2* and *sul3* (sulfonamides), and *tet(A)* (tetracycline) (Table 2). Whole-genome sequencing identified ARGs corresponding to several of the antimicrobial classes evaluated phenotypically, including β-lactams, quinolones, aminoglycosides, sulfonamides, and tetracyclines. The distribution of these determinants and the antimicrobial resistance phenotype for each sequenced isolate is presented in Supplementary Table S4. The genomic analysis was used as a complementary approach to document the presence and diversity of resistance determinants among the selected *E. coli* isolates, rather than to perform a complete genotype–phenotype association analysis.

## 4. Discussion

The Ring of Cenotes forms part of the Yucatán aquifer, which is particularly relevant because it constitutes the main freshwater source for the region and supports human activities. The characteristics of this aquifer, including high infiltration and limited natural filtration, increase its vulnerability to contamination from surface activities. The extensive hydraulic connectivity of the Ring of Cenotes may facilitate the movement of surface microorganisms and contaminants over relatively extended areas, making source attribution difficult in the absence of hydrological and land-use data. Previous studies in this area have reported antibiotic-resistant bacteria in groundwater, indicating that AMR is already present in this aquatic system. In the present study, we identified a widespread occurrence of culturable ampicillin-resistant Gram-negative bacteria across the sampling sinkholes. Members of the genera *Enterobacter*, *Escherichia*, and *Klebsiella* were the most frequently recovered bacteria across the multiple hydrogeological zones and sampling periods.

The occurrence of culturable ampicillin-resistant Gram-negative bacteria varied among hydrogeological zones and between sampling seasons. A larger number of isolates were recovered during the rainy season, and the site-positivity analysis also showed that several dominant genera, such as *Enterobacter*, *Escherichia*, and *Klebsiella*, were detected in a higher proportion during this season. These results suggest that seasonal hydrological processes may influence their occurrence within the aquifer. Seasonal recharge may contribute to these differences, since precipitation in karst systems can promote the movement of groundwater and surface-derived contaminants through the aquifer.

Similar temporal patterns have been reported in urban karst groundwater, where a higher occurrence and relative abundance of antimicrobial resistance genes were observed during periods of recharge, although the effect of the season was not always significant [20]. However, rainfall, groundwater flow, and other environmental variables were not measured in the present study, and their contribution to the observed seasonal differences cannot be established directly.

In addition to the dominant Enterobacterales, other Gram-negative bacteria were recovered, including opportunistic species such as *A. baumannii* and *P. aeruginosa*. Both species have also been recovered from different aquatic environments, showing that their occurrence is not restricted to clinical settings [21,22]. Other genera commonly associated with environmental and plant-related niches, including *Pantoea*, *Kosakonia*, and *Agrobacterium*, were also identified.

The presence of bacteria with different ecological associations in the same groundwater system reflects the complexity of the microbial populations recovered from the Ring of Cenotes. From a One Health perspective, this coexistence is relevant because environmental waters may provide interfaces where environmental bacteria and opportunistic pathogens occur together.

All isolates were recovered under ampicillin selection, while acquired resistance to additional antimicrobial classes was observed among several bacterial groups. Resistance to cefotaxime and tetracycline was detected in different species, whereas resistance to gentamicin and levofloxacin was less frequent. Sulfamethoxazole and nalidixic acid were also evaluated, although categorical susceptibility was not assigned because applicable CLSI criteria were unavailable. After excluding intrinsic or expected resistance, several bacterial genera showed acquired resistance involving more than one antimicrobial class. No acquired resistance to imipenem was detected, and therefore our results do not provide evidence of carbapenem-resistant populations in the isolates analyzed. The occurrence of resistance to different antimicrobial classes is consistent with previous studies in the Yucatán Peninsula. A diverse resistome, including determinants associated with tetracyclines, fluoroquinolones, β-lactams, cephalosporins, and aminoglycosides, has been reported in coastal environments of Yucatán, while antibiotic-resistant *E. coli* with resistance to multiple antimicrobial agents has recently been recovered from sinkholes of the Ring Cenotes Aquifer [13,23].

The recovery of opportunistic Gram-negative bacteria such as *A. baumannii, P. aeruginosa,* and several members of the Enterobacterales shows that the culturable ampicillin-resistant fraction of the aquifer included bacteria of clinical relevance. As a complementary component of the study, whole-genome sequencing was performed on a selected group of *E. coli* isolates to describe the resistance determinants present in this environmental bacterial population.

Whole-genome sequencing of the selected *E. coli* isolates identified resistance determinants associated with β-lactams, quinolones, aminoglycosides, sulfonamides, and tetracyclines. These findings provide complementary genomic evidence of the diversity of resistance determinants carried by environmental *E. coli* recovered from the aquifer. Previous studies from the Yucatán Peninsula have also reported ARGs associated with several antimicrobial classes in groundwater and coastal environments, while antibiotic-resistant *E. coli* with resistance to multiple antimicrobial agents have recently been recovered from sinkholes in this aquifer [13,23]. The presence of resistant bacteria carrying multiple resistance determinants in aquatic environments is relevant because water systems can act as reservoirs and routes for the dissemination of antimicrobial resistance genes among bacterial populations [24,25]. The resistance determinants identified by whole-genome sequencing were generally consistent with the antimicrobial classes in which resistance was observed phenotypically. However, the genomic component of this study was not designed to determine the complete genetic basis of each resistance phenotype. Other mechanisms, including chromosomal mutations, regulatory changes, gene expression, or intrinsic resistance determinants, may also contribute to the observed phenotypes but were not comprehensively evaluated.

The sequenced *E. coli* isolates exhibited considerable phylogenetic diversity, with the majority belonging to phylogroup B1 and the 19 isolates distributed among 16 sequence types. Most STs were represented by a single isolate, while ST1204, ST3759, and ST10068 were each detected in two isolates, indicating the absence of a predominant sequence type within the sequenced collection. Phylogroup B1 is frequently associated with environmental and animal populations, and freshwater-adapted *E. coli* are commonly represented within this phylogenetic group [26]. Together with the high diversity of STs and the absence of major ExPEC lineages commonly associated with human infections, these findings suggest that the resistant *E. coli* recovered from the sinkholes did not represent the expansion of a single dominant clinical lineage [27]. Instead, the genomic diversity observed is more consistent with a heterogeneous *E. coli* population within the aquifer.

From a One Health perspective, the Ring of Cenotes represents an important interface where environmental conditions and human, agricultural, and animal activities are closely connected. The recovery of opportunistic bacterial species, bacteria exhibiting resistance to multiple antimicrobial classes, and environmental microorganisms within the same groundwater system shows that this aquifer can harbor diverse bacterial populations relevant to environmental AMR [25,28]. Previous studies have also documented pesticide residues and other anthropogenic contaminants in the groundwater of the Yucatán Peninsula [29]. The coexistence of these contaminants with antimicrobial-resistant bacteria is relevant because non-antibiotic environmental stressors may contribute to the selection or maintenance of resistance through co-selection mechanisms [25,28].

Several limitations should be considered when interpreting these findings. Species identification was based primarily on MALDI-TOF MS using the manufacturer’s recommended confidence threshold. Although all reported identifications fulfilled this criterion, MALDI-TOF MS may not completely discriminate closely related members of the *Enterobacter cloacae* and *Klebsiella oxytoca* species complexes [30,31]. The study focused exclusively on culturable ampicillin-resistant Gram-negative bacteria and therefore does not represent the complete microbial diversity present within the aquifer. In addition, environmental variables such as land use, wastewater inputs, livestock density, and groundwater flow were not evaluated directly. Consequently, the specific sources and transport pathways of resistant bacteria could not be determined.

Overall, these findings demonstrate that the Ring of Cenotes aquifer harbors diverse populations of antibiotic-resistant Gram-negative bacteria, including opportunistic species and bacteria exhibiting diverse antimicrobial resistance profiles. Their occurrence across different hydrogeological zones and sampling periods shows that this groundwater system represents an important environmental compartment for antimicrobial resistance. These findings support the inclusion of vulnerable karst aquifers in environmental AMR surveillance and reinforce their relevance within a One Health framework.

## 5. Conclusions

This study shows that sinkholes of the Ring of Cenotes harbor diverse culturable populations of ampicillin-resistant Gram-negative bacteria distributed across different hydrogeological zones, including opportunistic pathogens and environmental bacteria with diverse antimicrobial resistance profiles. The higher occurrence of resistant bacteria during the rainy season suggests that hydrological conditions may influence their distribution within this karst system. The genomic analysis of selected *E. coli* isolates also revealed a highly diverse population, together with resistance determinants associated with several antimicrobial classes. These findings suggest that antimicrobial resistance in the Ring of Cenotes is present within a heterogeneous bacterial population rather than being associated with the spread of a single dominant lineage. Although the origin and mechanisms of dissemination of these bacteria were not investigated, our results support the role of karst groundwater as an environmental compartment for antimicrobial resistance and the relevance of these vulnerable aquifers to One Health surveillance.

## Figures and Tables

**Table 1:** Antimicrobial susceptibility matrix of the predominant culturable ampicillin-resistant Gram-negative bacteria recovered from the Ring of Cenotes aquifer. Values represent the number and percentage of isolates classified as resistant according to the applicable CLSI M100, 32nd edition (2022), interpretive criteria. IR indicates intrinsic or expected resistance, and NA indicates organism–antimicrobial combinations for which applicable CLSI interpretive criteria were unavailable. Only species represented by ≥3 isolates are shown. AMP, ampicillin; CTX, cefotaxime; GEN, gentamicin; LEV, levofloxacin; NAL, nalidixic acid; SMX, sulfamethoxazole; TET, tetracycline; IMI, imipenem.

Species	*n*	AMP	CTX	GEN	LEV	NAL	SMX	TET	IMI
*Enterobacter hormaechei*	29	IR	4 (13.8%)	0 (0%)	0 (0%)	NA	NA	1 (3.4)	0 (0)
*Escherichia coli*	26	26 (100%)	1 (3.8%)	0 (0%)	3 (11.5%)	NA	NA	7 (26.9)	0 (0)
*Klebsiella oxytoca*	12	IR	0 (0%)	0 (0%)	0 (0%)	NA	NA	0 (0)	0 (0)
*Kosakonia cowanii*	11	11 (100%)	2 (18.2%)	0 (0%)	0 (0%)	NA	NA	1 (9.1)	0 (0)
*Klebsiella pneumoniae*	10	IR	0 (0%)	0 (0%)	0 (0%)	NA	NA	1 (10)	0 (0)
*Citrobacter freundii*	7	IR	1 (14.3%)	1 (14.3%)	0 (0%)	NA	NA	1 (14.3)	0 (0)
*Pantoea dispersa*	7	7 (100%)	1 (14.3%)	0 (0%)	0 (0%)	NA	NA	0 (0)	0 (0)
*Acinetobacter baumannii*	6	NA	3 (50%)	0 (0%)	0 (0%)	NA	NA	0 (0)	0 (0)
*Proteus mirabilis*	6	6 (100%)	1 (16.7%)	0 (0%)	0 (0%)	NA	NA	5 (83.3)	0 (0)
*Enterobacter bugandensis*	5	IR	0 (0%)	0 (0%)	0 (0%)	NA	NA	0 (0)	0 (0)
*Klebsiella variicola*	5	IR	0 (0%)	0 (0%)	0 (0%)	NA	NA	0 (0)	0 (0)
*Pantoea anthophila*	5	5 (100%)	0 (0%)	0 (0%)	0 (0%)	NA	NA	0 (0)	0 (0)
*Acinetobacter pittii*	4	NA	2 (50%)	0 (0%)	0 (0%)	NA	NA	0 (0)	0 (0)
*Acinetobacter radioresistens*	4	NA	3 (75%)	0 (0%)	0 (0%)	NA	NA	0 (0)	0 (0)
*Leclercia adecarboxylata*	4	4 (100%)	1 (25%)	0 (0%)	0 (0%)	NA	NA	0 (0)	0 (0)
*Pseudescherichia vulneris*	4	4 (100%)	0 (0%)	0 (0%)	0 (0%)	NA	NA	0 (0)	0 (0)
*Serratia marcescens*	4	IR	0 (0%)	0 (0%)	0 (0%)	NA	NA	2 (50)	0 (0)
*Citrobacter braakii*	3	IR	1 (33.3%)	0 (0%)	0 (0%)	NA	NA	0 (0)	0 (0)
*Pseudomonas aeruginosa*	3	IR	IR	3 (100%)	0 (0%)	NA	IR	2 (66.7)	0 (0)

**Table 2:** Distribution of antimicrobial resistance determinants identified among the 19 sequenced *E. coli* isolates. Antimicrobial resistance determinants are grouped according to the corresponding antimicrobial class: β-lactams (*bla* genes), quinolones (*qnr* genes and mutations in *gyrA* and *parC*), aminoglycosides (*aadA* and *aph* genes), trimethoprim (*dfrA* genes), sulfonamides (*sul* genes), and tetracyclines (*tet* genes). Intrinsic resistance-associated determinants were excluded from the matrix. Colored cells indicate the presence of resistance genes while blank cells indicate that the determinant was not detected.

			Beta-Lactamase								Quinolones				Aminoglycoside					Trimethoprim			Sulfonamide		Tetracycline	
Genbank ID	Phylogroup	ST	*bla*Ec5	*bla*Ec8	*bla*Ec13	*bla*Ec14	*bla*Ec15	*bla*Ec18	*bla*TEM1	*bla*TEM135	*qnrs1*	*qnrb7*	*gyrA*	*parC*	*aadA*	*APH(3″)-Ib*	*APH(6)-Id*	*aadA2*	*aadA5*	*dfrA12*	*dfrA15*	*dfrA17*	*sul2*	*sul3*	*tet(A)*	*tet(B)*
**GCA_042599715**	**B1**	**937**																								
**GCA_042599975**	**E**	**3018**																								
**GCA_042600035**	**G**	**738**																								
**GCA_042600055**	**E**	**1204**																								
**GCA_042600075**	**B2**	**491**																								
**GCA_042600095**	**B1**	**13895**																								
**GCA_042600115**	**B1**	**3759**																								
**GCA_042603145**	**B1**	**3759**																								
**GCA_042603495**	**B1**	**16549**																								
**GCA_042603515**	**E**	**1204**																								
**GCA_055682845**	**B1**	**641**																								
**GCA_055682825**	**A**	**165**																								
**GCA_055682745**	**B1**	**2165**																								
**GCA_055682545**	**B2**	**136**																								
**SAMN62055834**	**A**	**746**																								
**SAMN62055833**	**D**	**362**																								
**GCA_055682965**	**B1**	**10068**																								
**GCA_055682275**	**B2**	**363**																								
**GCA_055682985**	**B1**	**10068**																								

## Data Availability

The original Genomic data presented in the study are openly available in GenBank at BioProject: PRJNA745210. The individual genome assembly accession numbers are: GCA_042599715, GCA_042599975, GCA_042600035, GCA_042600055, GCA_042600075, GCA_042600095, GCA_042600115, GCA_042603145, GCA_042603495, GCA_042603515, GCA_055682845, GCA_055682825, GCA_055682745, GCA_055682545, GCA_055682965, GCA_055682275, and GCA_055682985. The accession numbers for the sequenced isolates are provided in Supplementary Table S4.

## Supplementary Materials

The following supporting information can be downloaded at: https://www.mdpi.com/article/10.3390/microorganisms14091903/s1, Table S1. Geographic coordinates and hydrogeological classification of the 27 sinkholes sampled in the Ring of Cenotes aquifer, Yucatán, Mexico; Table S2. Organism-specific interpretive criteria used for antimicrobial susceptibility interpretation and multidrug-resistance classification; Table S3. Sequencing and genome assembly quality metrics for the 19 *Escherichia coli* isolates analyzed by whole-genome sequencing; Table S4. Isolate-level metadata, antimicrobial resistance phenotypes, resistance determinants, phylogroups, sequence types, and GenBank assembly accessions of the 19 *Escherichia coli* isolates selected for whole-genome sequencing.
